# Effect of ultrasonography and fluoroscopic guidance on the incidence of complications of cannulation in extracorporeal cardiopulmonary resuscitation in out-of-hospital cardiac arrest: a retrospective observational study

**DOI:** 10.1186/s12871-016-0293-z

**Published:** 2017-01-06

**Authors:** Masahiro Kashiura, Kazuhiro Sugiyama, Takahiro Tanabe, Akiko Akashi, Yuichi Hamabe

**Affiliations:** Emergency and Critical Care Center, Tokyo Metropolitan Bokutoh Hospital, 4-23-15 Kotobashi, Sumida-ku, Tokyo, 130-8575 Japan

**Keywords:** Cardiopulmonary Resuscitation, Catheterization, Extracorporeal Membrane Oxygenation, Fluoroscopy, Ultrasonography

## Abstract

**Background:**

It remains unclear which cannulation method is best in cases of extracorporeal cardiopulmonary resuscitation (ECPR) for out-of-hospital cardiac arrest. We assessed the effect of ultrasound- and fluoroscopy-guided percutaneous cannulation on complication incidence, compared with that using only ultrasound guidance.

**Methods:**

This single-center retrospective observational study was conducted between February 2011 and December 2015. In the comparison group, cannulation was performed percutaneously using only ultrasound guidance. In the exposure group, cannulation was performed percutaneously using fluoroscopy and ultrasound guidance. The primary outcome assessed was whether complications were associated with cannulation. The secondary outcome assessed was the duration from hospital arrival to extracorporeal circulation start. In addition to univariate analysis, multivariate logistic-regression analysis for cannulation complications was performed to adjust for several presumed confounders.

**Results:**

Of the patients who underwent ECPR, 73 were eligible; the comparison group included 50 cases and the exposure group included 23 cases. Univariate analysis showed that the complication incidence of the exposure group was significantly lower than that of the comparison group (8.7 vs. 36.0%, *p* = 0.022). Duration from hospital arrival to extracorporeal circulation start was almost the same in both groups (median, 17.0 min vs. 17.0 min, *p* = 0.92). After multivariate logistic regression analysis, cannulation using fluoroscopy and ultrasound was independently associated with a lower complication incidence (adjusted odds ratio, 0.14; *p* = 0.024).

**Conclusions:**

Ultrasound- and fluoroscopy-guided cannulation may reduce the complication incidence of cannulation without delaying extracorporeal circulation start.

**Electronic supplementary material:**

The online version of this article (doi:10.1186/s12871-016-0293-z) contains supplementary material, which is available to authorized users.

## Background

Out-of-hospital cardiac arrest (OHCA) is a major global public health concern. In the United States and Europe, approximately 330,000 and 275,000 individuals, respectively, develop OHCA annually [1, 2]. Despite the development of cardiopulmonary resuscitation (CPR), OHCA prognosis remains poor, with a survival rate of approximately 10% [1, 3, 4].

Extracorporeal membrane oxygenation (ECMO) is a technique for providing both cardiac and respiratory support to patients with cardiac or respiratory failure. Several observational studies showed that ECMO-assisted cardiopulmonary resuscitation, called extracorporeal cardiopulmonary resuscitation (ECPR), improved prognosis in special circumstances of OHCA [5–10]. The American Heart Association (AHA) and European Resuscitation Council (ERC) guidelines for CPR recommend considering ECMO in patients who have an easily reversible event and have had excellent CPR [11, 12].

Although ECPR may be beneficial for OHCA patients, there are many complications associated with cannulation, such as bleeding, ischemia of ipsilateral extremities, and vascular injury [13]. Approximately 10-20% of patients who underwent ECMO for cardiac and respiratory failure experienced complications from cannulation [13]. Moreover, while both ultrasound and fluoroscopy can facilitate ECMO cannulation in cases of cardiac or respiratory failure [14, 15], there is no reliable evidence to support which cannulation method is better in ECPR. The Extracorporeal Life Support Organization (ELSO) recommends a cut-down method for cannula insertion as the first choice in ECPR cases because of no pulsation at the femoral artery [16]. Percutaneous cannulation is only recommended in cases where access to the vessels exists prior to CPR [16]. However, the percutaneous cannulation method has the advantage of speed, which is a very important factor in OHCA prognosis [14].

This study aims to assess the effect of two percutaneous cannulation methods (using ultrasound and fluoroscopy versus using ultrasound alone) on complication incidence and time to ECMO start in ECPR for OHCA.

## Methods

### Setting and design

This retrospective before-after study was conducted in a single tertiary emergency medical center, which covered the local population of approximately 1,800,000 in the east Tokyo metropolitan area of Japan. While this institution established a new emergency room with fluoroscopic capability in August 2014, the distance from the ambulance parking area to the emergency room (approximately 50 m) did not change.

### Participants

The participants included were OHCA patients who underwent ECPR at the emergency department from 1 February 2011 to 31 December 2015. Patients were excluded if spontaneous circulation was restored before hospital arrival or between hospital arrival and ECMO start. The indication for ECPR has two criteria sets in our institution, as follows: (i) initial shockable rhythm, time from cardiac arrest to hospital arrival < 30 min, witnessed by bystander, and age < 65 years old, or (ii) witnessed by emergency medical service personnel, presumed reversible etiologies (e.g. cardiac disease, pulmonary embolism, incidental hypothermia, drug overdose), and age < 70 years old. Outside of these circumstances, ECPR is performed at the discretion of the emergency physician.

### ECPR protocol, techniques, and devices

ECPR implementation for OHCA occurred immediately after arrival at the emergency room. Both the outflow and inflow cannulas were inserted into contralateral or ipsilateral femoral vessels percutaneously using the Seldinger technique by emergency physicians. A blood circuit set, including a pump and membrane oxygenator, was primed using normal saline with 3,000 units of heparin. The ECMO pump flow rate was set between 3 and 4 L/min at the physician’s discretion. After ECMO start, a 4 French (Fr) sheath was inserted into a superficial femoral artery for delivering antegrade blood flow to a lower extremity.

The principal component of the ECMO circuit was a heparin-bonded surface circuit, including a centrifugal pump and hollow-fiber oxygenator (MERA CPB Circuit; Senko Medical Instrument Mfg. Corp., Tokyo, Japan or Capiox EBS; Terumo Corp., Tokyo, Japan). The ECMO circuit was connected to a heat exchanger to control body temperature. For the femoral artery, 15–16 Fr cannulas were used; 21–22 Fr cannulas were used for the femoral vein (Flexmate; Senko Medical Instrument Mfg. Corp., Tokyo, Japan or Capiox percutaneous catheter; Terumo Corp., Tokyo, Japan). Because of urgency for ECPR, cannula size selection was at the discretion of emergency physician without detailed measurement of vessel size.

All emergency physicians who were involved in ECPR underwent simulation training including cannulation methods, assistance of cannulation, operation of imaging devices, and priming the ECMO circuit before participating actual ECPR.

### Exposure and comparison group definition

Details of cannulation methods for the comparison and exposure groups are shown in the Supplementary Material [see Additional file 1: Figure S1]. From February 1, 2011, to July 31, 2014, cannulas were inserted in the femoral vessels using only ultrasound guidance in all patients. These patients were classified as the comparison group. The cannulation method was as follows: first, femoral vessels were punctured using ultrasound guidance with a linear type ultrasonic probe (HI VISION Avius; Hitachi Corp., Tokyo, Japan). Second, a guiding wire and cannula were inserted, confirming appropriate position (intra descending aorta or inferior vena cava) using a convex-type probe.

From August 1, 2014, to December 31, 2015, cannulas were inserted in the femoral vessels using ultrasound and fluoroscopy guidance. These patients were classified as the exposure group. The cannulation method was as follows: first, femoral vessels were punctured using a linear type ultrasonic probe, as in the comparison group. Second, a guiding wire was inserted to appropriate position using fluoroscopy (Infinix Celeve-i INFX-8000C; Toshiba Medical Systems Corp., Tochigi, Japan). When the punctured site was dilated, the direction of the dilator was matched with the direction of the guiding wire using fluoroscopy. Finally, the position of the outflow and inflow cannula was confirmed using fluoroscopy.

### Data collection

Data (patient demographics, arrest characteristics, and outcomes) of the two groups were extracted from electronic medical records retrospectively. We selected age, sex, anticoagulant or antiplatelet medication use, vascular disease, time period, and operators’ experience with cannulation in ECPR as potential confounders. Presence of vascular disease was defined as history of arteriosclerosis obliterans, arterial aneurysm, or arterial dissection. The details of history and medications, including anticoagulant and antiplatelet agents before admission, were obtained from patients’ relatives or family doctors. Time period in daytime was defined as patient transport to the hospital between 8 am and 6 pm. Operators’ experience level of cannulation in ECPR could not be obtained from medical records. Therefore, operators’ years of experience from involvement in ECPR cannulation was measured alternatively as a surrogate marker.

### Outcome measurements

The primary outcome assessed was complication associated with cannulation such as hematoma (subcutaneous or retroperitoneal), bleeding at the cannulation site, aberrant placement of cannula, vascular injury, or change to surgical approach. Hematoma (defined as > 3 cm) at the cannulation site, aberrant placement of cannula, and vascular injury were evaluated using computed tomography (Aquilion CX, TSX-101A; Toshiba Medical Systems Corp., Tochigi, Japan). Computed tomography was performed in all cases within two hours of starting ECMO, and coronary angiography, if necessary, was performed before or after computed tomography. In cases with possible cardiac causes, we usually performed coronary angiography before computed tomography. In OHCA cases with possible non-cardiac causes such as pulmonary embolism, incidental hypothermia, or drug overdose, for example, we first performed computed tomography. Bleeding was defined as obvious bleeding from a puncture site with transfusion requirement within 24 h of ECMO start. The secondary outcome assessed was the time from hospital arrival to ECMO pump start.

### Statistical analysis

Descriptive statistics were calculated for all variables of interest. Continuous variables were reported as medians and interquartile ranges, whereas categorical variables were summarized using counts and percentages. Univariate analysis was performed using Fisher’s exact test for categorical variables and Mann–Whitney *U* test for continuous variables. Multivariate logistic regression analysis was conducted to estimate the effect size of cannulation method using ultrasound and fluoroscopy on complication of cannulation after adjusting for potential confounders (age, sex, anticoagulant or antiplatelet medication use, vascular disease, time period, and operators’ years of experience). All reported *p* values were two-tailed, and values less than 0.05 were considered statistically significant. Statistical analysis was performed using IBM SPSS for Mac Version 22.0 (IBM Corp., Armonk, NY).

## Results

### Patient flowchart

**Fig. 1:**
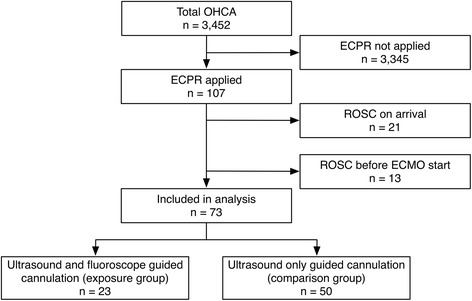
Flowchart of study patients who underwent ECPR. ECMO, extracorporeal membrane oxygenation; ECPR, extracorporeal cardiopulmonary resuscitation; OHCA, out-of-hospital cardiac arrest; ROSC, return of spontaneous circulation

Between February 2011 and December 2015, in total, 107 patients underwent ECPR. Of these, the study included 73 patients during the study period: 50 patients in the comparison group (42-month period) and 23 patients in the exposure group (17-month period) (Fig. 1).

### Baseline and cardiac arrest characteristics

The baseline characteristics of the two groups are summarized in Table 1. There was no significant difference between the two groups in presumed confounders (age, sex, anticoagulant or antiplatelet medication use, arrest in daytime, or comorbidity of vascular disease). Coronary angiography was more frequently performed in the exposure group.

**Table 1:** Baseline characteristics in out-of-hospital cardiac arrest patients who underwent extracorporeal cardiopulmonary resuscitation

	Overall (*n* = 73)	Ultrasound- and fluoroscopy-guided cannulation (*n* = 23)	Ultrasound only-guided cannulation (*n* = 50)
Age, years	56.0 [46.0, 64.0]	60.0 [53.0, 64.0]	55.0 [42.0, 62.0]
Male sex	63 (86)	19 (83)	44 (88)
Anticoagulant or antiplatelet medication use	15 (21)	7 (30)	8 (16)
Comorbidities			
Vascular disease	14 (19)	6 (26)	8 (16)
Cardiac disease	24 (33)	11 (48)	13 (26)
Time of day (8 am to 6 pm)	39 (53)	10 (44)	29 (58)
Prehospital variables			
Witness	59 (81)	20 (87)	39 (78)
Bystander CPR	40 (55)	15 (65)	25 (50)
Adrenaline administration	15 (21)	6 (26)	9 (18)
Definitive airway insertion	3 (4.1)	3 (13)	0 (0.0)
Shock delivery	62 (85)	19 (83)	43 (86)
ROSC during transportation	4 (5.5)	3 (13)	1 (2.0)
Time from call to hospital arrival, min	28.0 [23.0, 35.0]	28.0 [23.0, 33.0]	29.0 [24.0, 35.0]
Initial rhythm			
Ventricular fibrillation/Ventricular tachycardia	59 (81)	16 (70)	43 (86)
Pulseless electrical activity	12 (16)	5 (22)	7 (14)
Asystole	2 (2.7)	2 (8.7)	0 (0.0)
Rhythm on hospital arrival			
Ventricular fibrillation/Ventricular tachycardia	29 (40)	10 (44)	19 (38)
Pulseless electrical activity	24 (33)	8 (35)	16 (32)
Asystole	20 (27)	5 (22)	15 (30)
Operator's experience, yrs	2.0 [1.0, 3.0]	2.0 [1.0, 3.0]	3.0 [1.0, 3.0]
Subsequent interventions			
Antegrade flow cannula insertion	68 (93)	46 (92)	22 (96)
Coronary angiography	43 (59)	20 (87)	23 (46)
Percutaneous coronary intervention	24 (33)	11 (48)	13 (26)
Therapeutic hypothermia at 34° Celsius	34 (47)	14 (61)	20 (40)
Survival at discharge	14 (19)	5 (22)	9 (18)

CPR cardiopulmonary resuscitation; ROSC return of spontaneous circulation. Continuous variables are given as median [interquartile range, from 25th to 75th percentiles]. Categorical variables are given as count (percent)

### Univariate analysis of complication and time to ECMO start

The overall complication incidence was 27% (20/73). The complication incidence in the exposure group was significantly lower than that in the comparison group: 8.7% (2/23) vs. 36% (18/50), *p* = 0.022 (Table 2). Vascular injury, change to surgical approach, and aberrant placement of cannula were not observed in the exposure group. Duration from hospital arrival to extracorporeal circulation start was similar in the two groups (median, 17.0 min vs. 17.0 min, *p* = 0.92) (Table 2).

**Table 2:** Outcome in out-of-hospital cardiac arrest patients who underwent extracorporeal cardiopulmonary resuscitation

	Overall (*n* = 73)	Ultrasound- and fluoroscopy-guided cannulation (*n* = 23)	Ultrasound only-guided cannulation (*n* = 50)	*p* value
Complication associated with cannulation	20 (27)	2 (8.7)	18 (36)	0.022
Bleeding	6 (8.2)	1 (4.3)	5 (10)	0.66
Vascular injury	3 (4.1)	0 (0.0)	3 (6.0)	0.55
Change to surgical approach	4 (5.6)	0 (0.0)	4 (8.0)	0.30
Aberrant placement of cannula	3 (4.1)	0 (0.0)	3 (6.0)	0.55
Hematoma	15 (21)	2 (8.7)	13 (26)	0.12
Time from hospital arrival to ECMO start, minutes	17.0 [13.0, 23.0]	17.0 [14.0, 22.0]	17.0 [13.0, 25.0]	0.92
Time from call to ECMO start, minutes	46.0 [40.0, 55.0]	45.0 [38.0, 51.0]	46.0 [42.0, 56.0]	0.23

ECMO extracorporeal membrane oxygenation. Continuous variables are given as median [interquartile range, from 25th to 75th percentiles]. Categorical variables are given as count (percent)

### Multivariate logistic regression analysis for cannulation complication

After multivariate logistic regression analysis to estimate the effect of cannulation method and eliminate the effects of possible confounders, the combined method of cannulation (ultrasound- and fluoroscopy-guided) was consistently an independent factor associated with successful cannulation without complication (adjusted odds ratio: 0.14, 95% confidence interval: 0.02–0.77, *p* = 0.024) (Table 3).

**Table 3:** Multivariate logistic regression analysis of predictors for complication associated with cannulation in extracorporeal cardiopulmonary resuscitation

	Adjusted odds ratio (95% CI)	*p* value
Age, per year	1.00 (0.96–1.05)	0.92
Male sex	0.55 (0.09–3.51)	0.53
Anticoagulant or antiplatelet medication use	0.37 (0.06–2.10)	0.26
Comorbidity of vascular disease	1.28 (0.27–6.01)	0.76
Time period (8 am to 6 pm)	0.73 (0.22–2.44)	0.60
Operator's experience, per year	0.67 (0.45–1.01)	0.058
Ultrasound- and Fluoroscopy-guided cannulation	0.14 (0.02–0.77)	0.024

CI confidence interval

## Discussion

We investigated the effect of ultrasound- and fluoroscopy-guided cannulation on the incidence of complications and time to ECMO start in ECPR for OHCA patients in a single-center retrospective observational study. Ultrasound- and fluoroscopy-guided cannulation did not delay the time to ECMO start and was associated with lower incidence of complication even after adjusting for several confounders.

The combination of ultrasound- and fluoroscopy-guided cannulation was associated with reduced complication incidence. For respiratory or heart failure, cannulation complication incidence in veno-arterial or veno-venous ECMO was approximately 10–20% in previous studies [13, 17–21], whereas the incidence of complication for ECPR cannulation was approximately 25–60% [5, 22–25], which is relatively high. This difference in complication incidence probably results from urgency associated with cannulation in ECPR, unlike other indications. In our study, the overall incidence of complications was 27%, which is similar to that seen in previous studies [5, 22–25]. Moreover, percutaneous cannulation with ultrasound and fluoroscopic guidance appears to be a safer approach in ECPR cases, with only a 10% complication incidence. Ultrasound can allow secure puncture of femoral vessels, while fluoroscopy can prevent aberration and kinking of the guiding wire at the transparent cannulation site when the puncture site is dilated [14]. These points indicate that ultrasound- and fluoroscopy-guided cannulation contributed to reduce the incidence of complications.

Time from hospital arrival to ECMO circuit start was almost the same in the two groups. Survival rate of OHCA decreases as each minute passes [26]; therefore, time to initiation of ECMO circuit is a very important prognostic factor in ECPR [6, 24, 27, 28]. Mean time from hospital arrival to implementation of ECMO varied from 19 to 40 min in previous studies [5, 22, 29], whereas the median time was 17 min in both groups in the present study. A percutaneous approach with fluoroscopy and ultrasound is more complicated due to device operation. However, this complexity and potential risk of delay in cannulation seems to be reduced or eliminated by reducing the number of punctures and increasing the rapid and smooth dilation of the cannulation site [30]. Percutaneous cannulation with ultrasound and fluoroscopy guidance took less time in ECPR than percutaneous cannulation with ultrasound alone.

In the present study, only a percutaneous approach cannulation method was evaluated, despite ELSO recommendations [16]. However, a percutaneous approach is more often utilized, according to previous studies [5, 21, 22, 24, 31, ]. The Seldinger technique is adapted for insertion of large-diameter cannulas through the sequential use of several dilators of increasing size. The main advantage is a reduced risk of bleeding, and disadvantages include potential risk for displacement of a guiding wire, resulting in vessel injury or perforation, and difficulty in ensuring intraluminal placement [14]. Moreover, performing cannulation under resuscitation conditions is a major challenge. Detecting the target vessels under hypodynamic circulatory conditions is much more difficult [30]. Imaging modalities, including ultrasound and fluoroscopy, can overcome most of the limitations of a percutaneous approach [14]. Ultrasound can be used to determine vessel location and fluoroscopy can allow detection of incorrect placement of a guiding wire and cannula before perforation or other injury occurs. Thus, our practice includes using imaging during all phases of cannulation whenever possible, including vascular access, guiding wire insertion, and cannula placement.

The present study has several limitations. First, confounders such as coagulability and platelet count were not adjusted for, as these laboratory tests were performed after ECMO initiation. Since these laboratory test results were obtained after heparin administration and a centrifugal pump start, coagulability and platelet count were probably affected. Therefore, we adjusted anticoagulant or antiplatelet medication use as a surrogate marker of coagulability. Second, the number of physicians involved in ECPR, which might influence the outcome, could not be obtained from electronic medical records. We attempted to adjust for this possible confounder using time period, which is associated with the number of physicians. Third, complications associated with cannulation and time to ECMO start, not survival or neurologic outcome, were the outcomes assessed. However, bleeding complications surrounding ECMO are a major problem in managing post-cardiac arrest syndrome. Patients who experience cardiac arrest face several bleeding risks such as post-cardiac arrest syndrome, therapeutic hypothermia, and anticoagulation therapy [32–34]. Thus, successful cannulation without complication is an important and relevant outcome in managing OHCA patients with ECPR. Lastly, this study is a retrospective before-after study. Management of post-cardiac arrest syndrome may have changed in the elapsed time. At the time that this study was conducted, 2010 AHA or ERC CPR guidelines were recommended, and we consistently treated patients with OHCA based on those guidelines. In addition, physician skills might influence our results. Further investigation for seeking the best cannulation method for ECPR is needed.

## Conclusion

In ECPR for OHCA patients, a combination of ultrasound- and fluoroscopy-guided cannulation is associated with a lower incidence of cannulation complication than ultrasound only-guided cannulation, without delay in extracorporeal circulation start. This method can contribute to safer management of post-cardiac arrest syndrome.

## Additional file

Additional file 1: Figure S1. Cannulation method details of the comparison and exposure group. (PDF 8931 kb)

## Abbreviations

AHA: American Heart Association
CPR: Cardiopulmonary resuscitation
ECMO: Extracorporeal membrane oxygenation
ECPR: Extracorporeal cardiopulmonary resuscitation
ELSO: Extracorporeal Life Support Organization
ERC: European Resuscitation Council
Fr: French
OHCA: Out-of-hospital cardiac arrest

## References

[1] Berdowski J, Berg RA, Tijssen JGP, Koster RW (2010). Global incidences of out-of-hospital cardiac arrest and survival rates: Systematic review of 67 prospective studies. Resuscitation.

[2] Mozaffarian D, Benjamin EJ, Go AS, Arnett DK, Blaha MJ, Cushman M (2015). Heart disease and stroke statistics--2015 update: a report from the American Heart Association. Circulation.

[3] Sasson C, Rogers MAM, Dahl J, Kellermann AL (2010). Predictors of survival from out-of-hospital cardiac arrest: a systematic review and meta-analysis. Circ Cardiovasc Qual Outcomes.

[4] Kitamura T, Iwami T, Kawamura T, Nitta M, Nagao K, Nonogi H (2012). Nationwide improvements in survival from out-of-hospital cardiac arrest in Japan. Circulation.

[5] Kagawa E, Inoue I, Kawagoe T, Ishihara M, Shimatani Y, Kurisu S (2010). Assessment of outcomes and differences between in- and out-of-hospital cardiac arrest patients treated with cardiopulmonary resuscitation using extracorporeal life support. Resuscitation.

[6] Le Guen M, Nicolas-Robin A, Carreira S, Raux M, Leprince P, Riou B (2011). Extracorporeal life support following out-of-hospital refractory cardiac arrest. Crit Care.

[7] Wang C-H, Chou N-K, Becker LB, Lin J-W, Yu H-Y, Chi N-H (2014). Improved outcome of extracorporeal cardiopulmonary resuscitation for out-of-hospital cardiac arrest--a comparison with that for extracorporeal rescue for in-hospital cardiac arrest. Resuscitation.

[8] Sakamoto T, Morimura N, Nagao K, Asai Y, Yokota H, Nara S (2014). Extracorporeal cardiopulmonary resuscitation versus conventional cardiopulmonary resuscitation in adults with out-of-hospital cardiac arrest: a prospective observational study. Resuscitation.

[9] Kim SJ, Kim HJ, Lee HY, Ahn HS, Lee SW (2016). Comparing extracorporeal cardiopulmonary resuscitation with conventional cardiopulmonary resuscitation: A meta-analysis. Resuscitation.

[10] Ortega-Deballon I, Hornby L, Shemie SD, Bhanji F, Guadagno E (2016). Extracorporeal resuscitation for refractory out-of-hospital cardiac arrest in adults: A systematic review of international practices and outcomes. Resuscitation.

[11] Link MS, Berkow LC, Kudenchuk PJ, Halperin HR, Hess EP, Moitra VK (2015). Part 7: Adult Advanced Cardiovascular Life Support: 2015 American Heart Association Guidelines Update for Cardiopulmonary Resuscitation and Emergency Cardiovascular Care. Circulation.

[12] Soar J, Nolan JP, Böttiger BW, Perkins GD, Lott C, Carli P (2015). European Resuscitation Council Guidelines for Resuscitation 2015: Section 3. Adult advanced life support. Resuscitation.

[13] Bisdas T, Beutel G, Warnecke G, Hoeper MM, Kuehn C, Haverich A (2011). Vascular complications in patients undergoing femoral cannulation for extracorporeal membrane oxygenation support. Ann Thorac Surg.

[14] Conrad SA, Grier LR, Scott LK, Green R, Jordan M (2015). Percutaneous cannulation for extracorporeal membrane oxygenation by intensivists: a retrospective single-institution case series. Crit Care Med.

[15] Extracorporeal Life Support Organization. ELSO General Guidelines for all ECLS Cases. Version 1.3. Extracorporeal Life Support Organization. 2013. https://www.elso.org/Portals/0/IGD/Archive/FileManager/929122ae88cusersshyerdocumentselsoguidelinesgeneralalleclsversion1.3.pdf. Accessed 30 June 2016.

[16] Extracorporeal Life Support Organization. ELSO Guidelines for ECPR Cases. Version 1.3. Extracorporeal Life Support Organization. 2013. http://www.elso.org/Portals/0/IGD/Archive/FileManager/6713186745cusersshyerdocumentselsoguidelinesforecprcases1.3.pdf. Accessed 30 June 2016.

[17] Burrell AJC, Pellegrino VA, Sheldrake J, Pilcher DV (2015). Percutaneous cannulation in predominantly venoarterial extracorporeal membrane oxygenation by intensivists. Crit Care Med.

[18] Australia and New Zealand Extracorporeal Membrane Oxygenation (ANZ ECMO) Influenza Investigators (2009). Extracorporeal membrane oxygenation for 2009 influenza a(H1N1) acute respiratory distress syndrome. JAMA.

[19] Xie A, Phan K, Tsai Y-C, Yan TD, Forrest P (2015). Venoarterial extracorporeal membrane oxygenation for cardiogenic shock and cardiac arrest: a meta-analysis. J Cardiothorac Vasc Anesth.

[20] Guttendorf J, Boujoukos AJ, Ren D, Rosenzweig MQ, Hravnak M (2014). Discharge outcome in adults treated with extracorporeal membrane oxygenation. Am J Crit Care.

[21] Aziz F, Brehm CE, El-Banyosy A, Han DC, Atnip RG, Reed AB (2014). Arterial complications in patients undergoing extracorporeal membrane oxygenation via femoral cannulation. Ann Vasc Surg.

[22] Kim SJ, Jung JS, Park JH, Park JS, Hong YS, Lee SW (2014). An optimal transition time to extracorporeal cardiopulmonary resuscitation for predicting good neurological outcome in patients with out-of-hospital cardiac arrest: a propensity-matched study. Crit Care.

[23] Maekawa K, Tanno K, Hase M, Mori K, Asai Y (2013). Extracorporeal cardiopulmonary resuscitation for patients with out-of-hospital cardiac arrest of cardiac origin: a propensity-matched study and predictor analysis. Crit Care Med.

[24] Haneya A, Philipp A, Diez C, Schopka S, Bein T, Zimmermann M (2012). A 5-year experience with cardiopulmonary resuscitation using extracorporeal life support in non-postcardiotomy patients with cardiac arrest. Resuscitation.

[25] Thiagarajan RR, Brogan TV, Scheurer MA, Laussen PC, Rycus PT, Bratton SL (2009). Extracorporeal membrane oxygenation to support cardiopulmonary resuscitation in adults. Ann Thorac Surg.

[26] Goto Y, Funada A, Goto Y. Relationship between the duration of cardiopulmonary resuscitation and favorable neurological outcomes after Out-of-hospital cardiac arrest: a prospective, nationwide, population-based cohort study. J Am Heart Assoc. 2016;4. 10.1161/JAHA.115.002819 PMC4943259 26994129

[27] Chen Y-S, Chao A, Yu H-Y, Ko W-J, Wu I-H, Chen RJ-C (2003). Analysis and results of prolonged resuscitation in cardiac arrest patients rescued by extracorporeal membrane oxygenation. J Am Coll Cardiol.

[28] Huang S-C, Wu E-T, Chen Y-S, Chang C-I, Chiu I-S, Wang S-S (2008). Extracorporeal membrane oxygenation rescue for cardiopulmonary resuscitation in pediatric patients. Crit Care Med.

[29] Lamhaut L, Jouffroy R, Soldan M, Phillipe P, Deluze T, Jaffry M (2013). Safety and feasibility of prehospital extra corporeal life support implementation by non-surgeons for out-of-hospital refractory cardiac arrest. Resuscitation.

[30] Swol J, Belohlavek J, Haft JW, Ichiba S, Lorusso R, Peek GJ (2016). Conditions and procedures for in-hospital extracorporeal life support (ECLS) in cardiopulmonary resuscitation (CPR) of adult patients. Perfusion.

[31] Johnson NJ, Acker M, Hsu CH, Desai N, Vallabhajosyula P, Lazar S (2014). Extracorporeal life support as rescue strategy for out-of-hospital and emergency department cardiac arrest. Resuscitation.

[32] Jacob M, Hassager C, Bro-Jeppesen J, Ostrowski SR, Thomsen JH, Wanscher M (2015). The effect of targeted temperature management on coagulation parameters and bleeding events after out-of-hospital cardiac arrest of presumed cardiac cause. Resuscitation.

[33] White NJ, Leong BS-H, Brueckner J, Martin EJ, Brophy DF, Peberdy MA (2011). Coagulopathy during cardiac arrest and resuscitation in a swine model of electrically induced ventricular fibrillation. Resuscitation.

[34] Adrie C, Monchi M, Laurent I, Um S, Yan SB, Thuong M (2005). Coagulopathy after successful cardiopulmonary resuscitation following cardiac arrest. J Am Coll Cardiol.

