# Supplementary material for: Effect of ultrasonography and fluoroscopic guidance on the incidence of complications of cannulation in extracorporeal cardiopulmonary resuscitation in out-of-hospital cardiac arrest: a retrospective observational study
Source: BMC Anesthesiol. 2017 Jan 6;17:4. doi: 10.1186/s12871-016-0293-z (PMC5267374; doi:10.1186/s12871-016-0293-z)

Supplementary material. Cannulation method details of two groups.

Comparison group (ultrasound guided percutaneous cannulation)

1. Femoral vessels are punctured by an 18G puncture needle while confirming with linear-type probe, in parallel with standard cardiopulmonary resuscitation continued as possible.

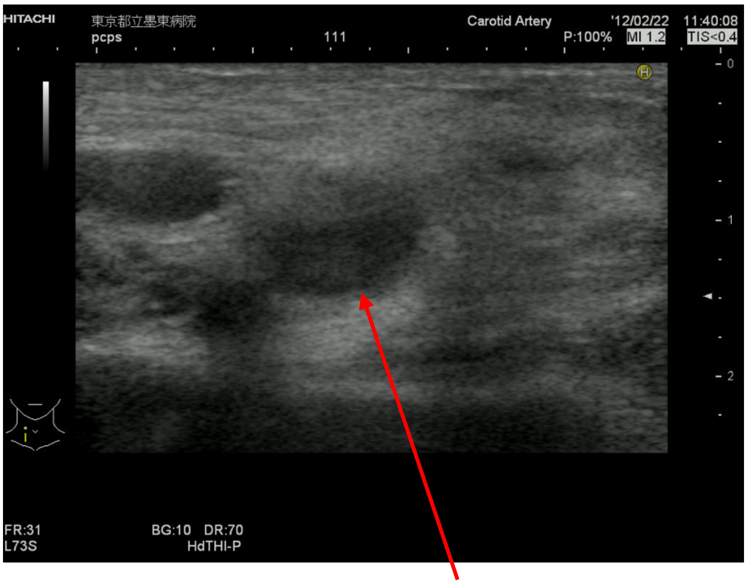

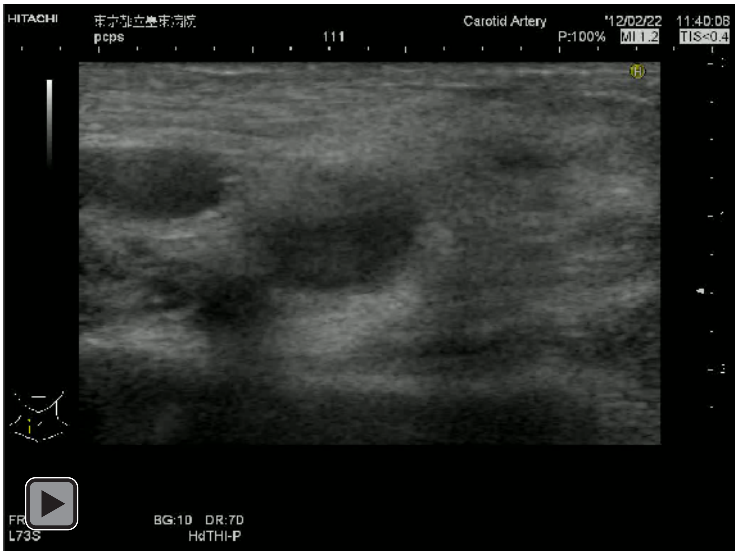

Femoral vein

Video (requirement of Acrobat Reader 9 or later)

2. Guiding wire and cannula were inserted to appropriate position using sector type probe.

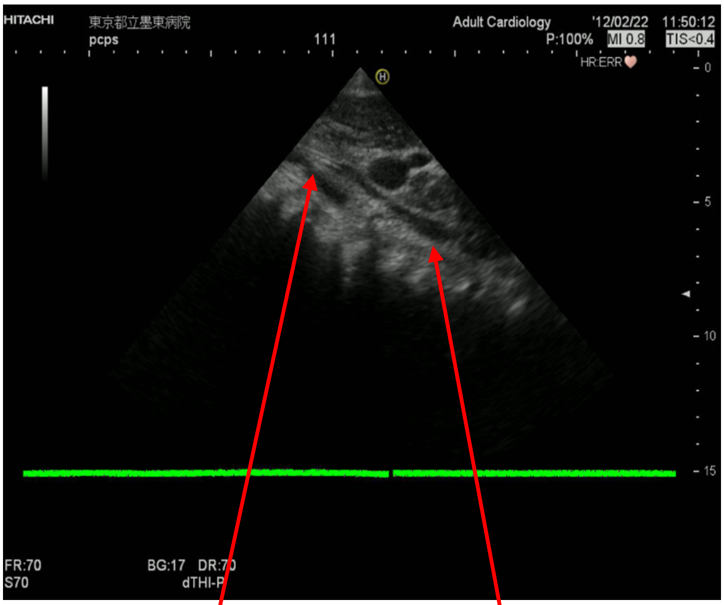

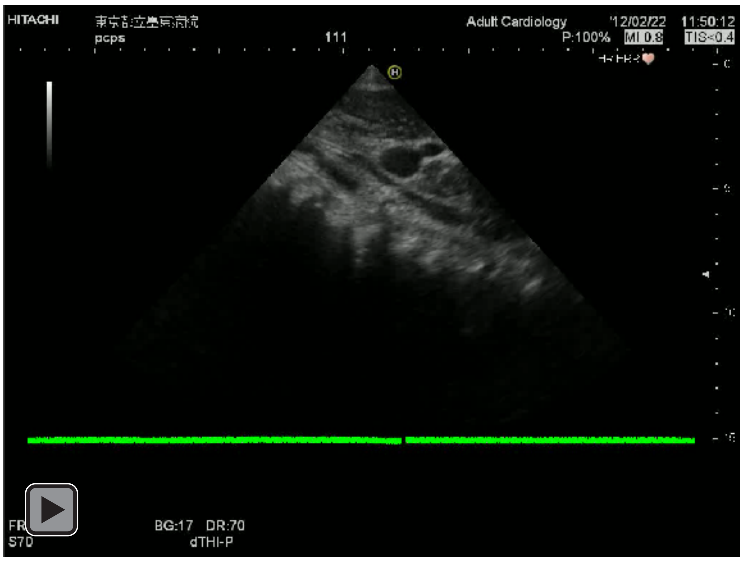

Guiding wire **Inferior vena cava**

Video (requirement of Acrobat Reader 9 or later)

Exposure group (ultrasound and fluoroscopy guided percutaneous cannulation)

1. Femoral vessels are punctured using linear type ultrasonic probe as with comparison group.

2. Guiding wire is inserted to appropriate position using fluoroscopy.

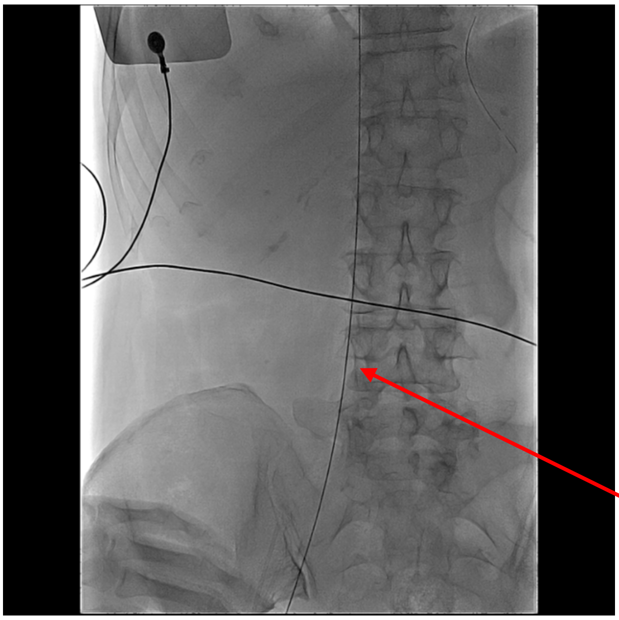

Guiding wire

3. The punctured site is dilated using a dilator; the direction of the dilator was matched with the direction of the guiding wire using fluoroscopy.

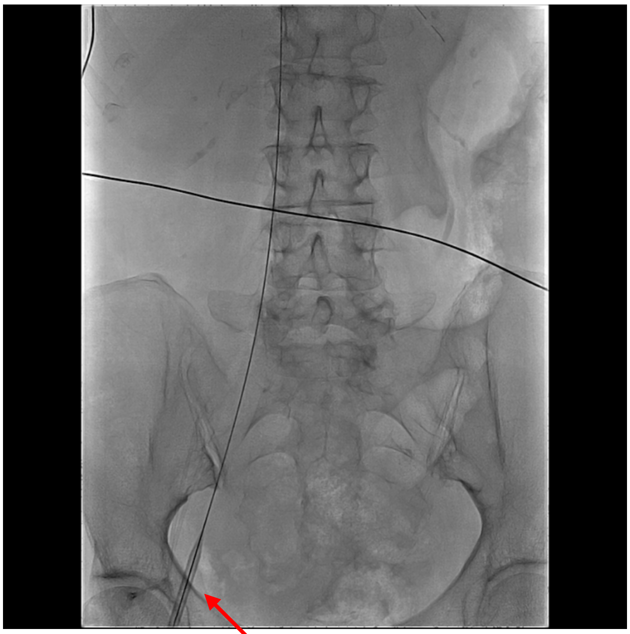

Appropriate dilator direction

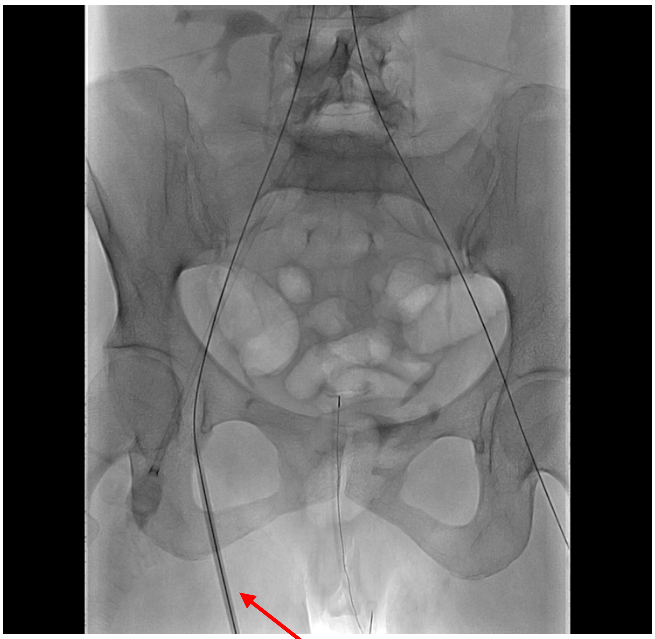

Wrong dilator direction

4. The position of cannula is confirmed using fluoroscopy.

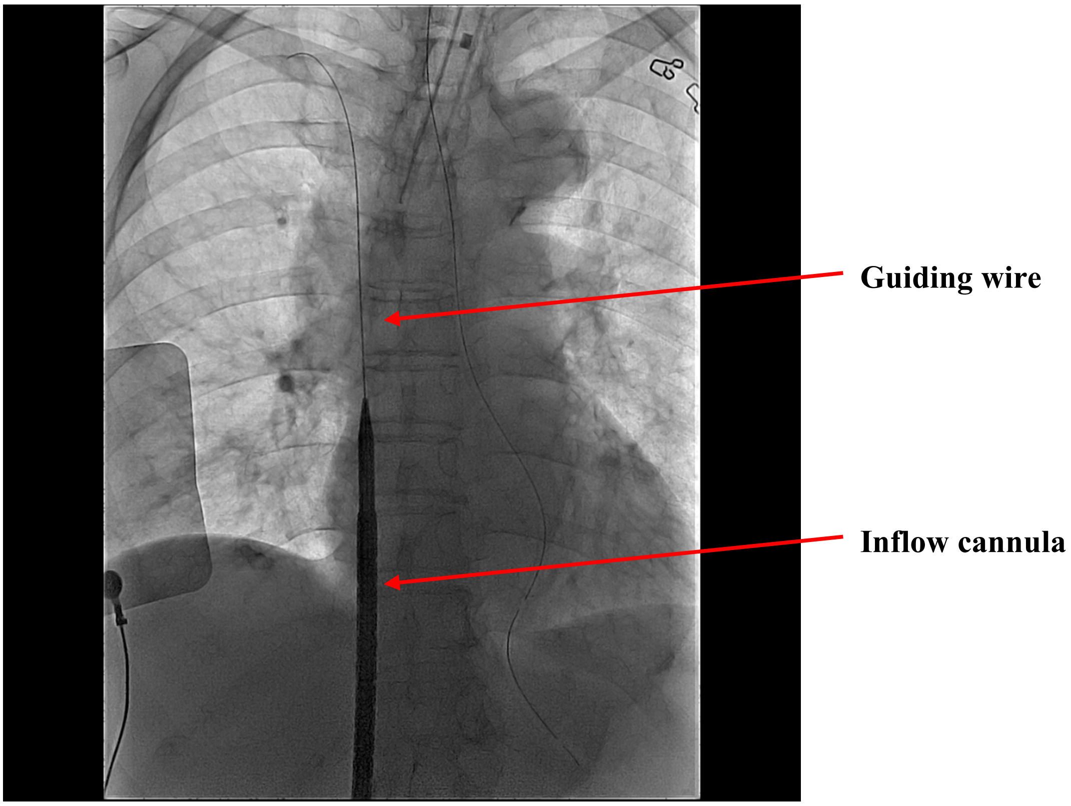

Supplement: Additional file 1: Figure S1. — Cannulation method details of the comparison and exposure group. (PDF 8931 kb) [file 12871_2016_293_MOESM1_ESM.pdf]
